# Study protocol for an open-label, single-arm, phase Ib/II study of combination of toripalimab, nab-paclitaxel, and gemcitabine as the first-line treatment for patients with unresectable pancreatic ductal adenocarcinoma

**DOI:** 10.1186/s12885-020-07126-3

**Published:** 2020-07-09

**Authors:** Lin Shui, Ke Cheng, Xiaofen Li, Pixian Shui, Xiaohan Zhou, Jian Li, Cheng Yi, Dan Cao

**Affiliations:** 1 Department of Abdominal Oncology, Cancer Center, West China Hospital, Sichuan University, Chengdu, China; 2 School of Pharmacy, Southwest Medical University, Luzhou, China; 3 Department of Pharmacy, The Affiliated Traditional Chinese Medicine Hospital of Southwest Medical University, Luzhou, China

**Keywords:** Pancreatic ductal adenocarcinoma, PD-1 blockade, Combination therapy, Clinical protocol

## Abstract

**Background:**

Pancreatic ductal adenocarcinoma (PDAC) is a fatal disease with a dismal response to single-use of immune checkpoint inhibitors (ICIs). ICIs combined with systemic therapy has shown efficacy and safety in various solid tumors. Nab-paclitaxel and gemcitabine (AG), as the standard first-line treatment for advanced PDAC, has been widely used in recent years. The combination of ICIs and AG chemotherapy appears to be a promising option in the treatment of PDAC.

**Methods:**

This is an open-label, single-arm, and single-center phase Ib/II trial. The enrolled subjects are the unresectable (locally advanced or metastatic) PDAC patients without previous systemic treatments. All subjects receive an intravenous injection of gemcitabine 1000 mg/m^2^ and nab-paclitaxel 125 mg/m^2^ on day 1 and day 8, along with toripalimab 240 mg at day 1 every 3 weeks. The subjects may discontinue the treatment because of progression disease (PD), intolerable toxicities, requirements of patients or researchers. For local advanced patients who are evaluated as partial response (PR), surgeons need to assess the surgical possibility. The primary objective of this trial is to evaluate the safety and overall survival (OS) of this combination therapy; and the secondary objective is related to the assessment of objective response rate (ORR), disease control rate (DCR), progression-free survival (PFS), and the rate of resection or R0 resection after receiving toripalimab plus AG treatment. Besides, we expect to identify the predictive biomarkers (such as MMR protein and PD-L1 expression, the number of TILs, the small RNA of EBV and so on) and explore the correlation between these biomarkers and tumor response to this combined regimen.

**Discussion:**

This trial is the first attempt to evaluate the efficacy and safety of the combination of toripalimab plus AG chemotherapy as a first-line treatment for unresectable PDAC patients. The results of this phase Ib/II study will provide preliminary evidence for further assessment of this combined therapeutic regimen for unresectable PDAC patients.

**Trial registration:**

Trial registration: ChiCTR (ChiCTR2000032293). Registered 25 April 2020 - Retrospectively registered.

## Background

Pancreatic ductal adenocarcinoma (PDAC) is a highly aggressive tumor with high mortality. Due to its insidious onset, a significant part of patients is initially diagnosed as locally advanced or metastatic with a 5-year survival rate of less than 9% [1]. Recently, the emergence of chemotherapy regimens, such as gemcitabine combined with nab-paclitaxel (AG) or FOLFIRINOX, improved the survival of patients with the late-stage PDAC [2, 3]. Given the dismal overall prognosis, novel treatment strategies are urgently needed to further reform the treatment for unresectable PDAC.

Immune checkpoint inhibitor (ICI) is an established treatment and approved for multiple solid tumors. ICIs enhance the anti-tumor immune response through stimulating tumor-specific T cells, thus eliminating tumor cells and generating durable immune memory. Programmed death-1 (PD-1) is a receptor of the immunoglobulin family expressed on the surface of activated T lymphocytes. PD-1 binds to its ligand PD-L1 (B7-H1) to mediate the inhibitory signal of immune response and plays a critical role to regulate the peripheral tolerance and the mechanism of immunosuppression or immune escape of tumor cells. PD-1/PD-L1 targeting therapy has become one of the important advances in changing treatment decisions in a variety of solid tumors. However, the success of PD-1/PD-L1 blockades was not replicated in PDAC. The single-use of PD-1 antibody has not shown objective response in metastatic PDACs [4]. The recent studies intended to improve the disappointing results of ICI therapy, and increasing evidence indicated the synergistic function of PD-1 antibody and other systemic therapy. Pembrolizumab combined with chemotherapy significantly improved the objective effective rate and overall survival of non-small cell lung cancer (NSCLC) patients compared with chemotherapy alone, with good tolerance and manageable side effects [5, 6]. The phase I studies of pembrolizumab combined with nab-paclitaxel and gemcitabine chemotherapy (AG) have shown good safety and efficacy [7], including those in digestive tract tumors [8, 9].

Besides, the potential population with favorable response to PD-1/PD-L1 blockades remains controversial. According to ASCO clinical practice guideline, PD-1 blockades are recommended for patients with high microsatellite instability (MSI-H) [10]. However, the incidence of MSI-H in PDAC is relatively low. Besides MSI, PD-L1 expression and tumor mutation burden (TMB) level [11–16] are also considered to potentially predict the response to PD-1 antibody. The results of this connection need to be confirmed in different tumor types and different immunotherapeutic drugs. Furthermore, the diversity of T cell receptors (TCRs) in peripheral blood, the cloning of TCR in tumor tissue and the number of tumor-infiltrating lymphocytes (TILs) are also regarded as possible predictive biomarkers [17, 18].

In summary, given the impressive efficacy of AG chemotherapy and the good tolerance of PD-1 blockades combined with AG showed in a phase I study [13], we conduct a phase Ib/II study enrolling 54 patients with unresectable (locally advanced or metastatic) PDAC to explore the efficacy and safety of toripalimab (a PD-1 monoclonal antibody) plus AG. Mismatch repair (MMR) protein, PD-L1 expression, the subset of T cells in peripheral blood, the TIL numbers and diversity of TCRs are estimated to identify the potential biomarkers for predicting the efficacy of the combined regimen. This trial is expected to preliminarily indicate the feasibility of the combined therapeutic approach as the first-line treatment in advanced PDAC patients and provide evidence for further research.

## Methods/design

### Design

This is an open-label, single-arm, and single-center phase Ib/II trial. The enrolled subjects are the unresectable (locally advanced or metastatic) PDAC patients without previous systemic treatments. All subjects receive an intravenous injection of gemcitabine 1000 mg/m^2^ and nab-paclitaxel 125 mg/m^2^ on day 1 and day 8, along with toripalimab 240 mg on day 1 every 3 weeks. After the patients sign the written informed consent, the treatment is administrated according to the protocol until the presence of disease progression (PD) or intolerable adverse events. In the case of grade 3 or higher adverse events (AEs), the treatment should be suspended and the AEs should be actively treated until returning to normal or grade 1 or 2. The next cycle of treatment may reduce the dose according to the decision of researchers. In addition, the medical safety team will review all safety information during this clinical study.

### Research hypothesis

The combination therapy of toripalimab plus GA as the first-line treatment prolongs the survival of patients with metastatic PDAC with good safety.

### Objectives

#### Primary objectives

To evaluate the safety and overall survival (OS) of first-line treatment with toripalimab and AG chemotherapy (nab-paclitaxel plus gemcitabine) in patients with locally advanced or metastatic pancreatic cancer without previous systemic treatments. Safety evaluation includes the patients’ tolerance to this regimen and the influence of these drugs on the skin, digestive tract, and electrolytes balance of patients.

#### Secondary objectives

The evaluation of objective response rate (ORR), disease control rate (DCR), progression-free survival (PFS), and the rate of surgical conversion and R0 resection for toripalimab combined with AG chemotherapy in patients with locally advanced or metastatic pancreatic cancer without previous systemic treatment.

#### Exploratory objectives

The exploratory objectives of this trial are to further investigate the predictive biomarkers for the efficacy of the combination therapy. The following parameters of patients are regarded as potential predictive biomarkers and the correlation between them and tumor response is explored.

1) In the tumor issues: the expression of DNA mismatch repair (MMR) protein and PD-L1, the number of TILs, and the small RNA of Epstein-Barr Virus (EBV);

2) In the peripheral blood: T cell subsets (the absolute counts of CD3, CD4, and CD8), the tumor markers, the heat shock protein 90 α, EBV-EAD (early antigen)-IgG, and EBV-VCA (viral capsid antigen)-IgA;

The following parameters of patients are estimated to predict the clinical outcomes and prognosis of this combination therapy: the TMB level results from exome sequencing of ctDNA in the tumor tissue or peripheral blood, the diversity of TCR in peripheral blood, and the cloning of TCR in the tumor tissue.

### Key eligibility criteria

The included patients are treatment-naive and unresectable (locally advanced or metastatic) patients with histopathologically confirmed PDAC. Besides, patients with an Eastern Cooperative Oncology Group performance status (ECOG PS) 0 to 2, adequate organ function, no history of active autoimmune disease, and no treatment history of ICI or chemotherapy, are eligible for this trial (detailed key inclusion and exclusion criteria are listed in Table 1).

**Table 1:** The key eligible criteria of this trial

Key inclusion and exclusion criteria	
Inclusion criteria	Exclusion criteria
Age between 18 and 80 years	Synchronous or metachronous (within 5 years) malignancies
Unresectable locally advanced or metastatic pancreatic cancer that is pathologically diagnosed as adenocarcinoma	Females who are pregnant, or lactating
No prior anti-tumor treatment for pancreatic cancer	New known or suspected uncontrolled metastases to brain
ECOG PS: 0 to 2	Serious or uncontrolled infectious disease (HIV、active tuberculosis、HBV DNA>103/ml)
Life expectancy ≥3 months	Active autoimmune disease requiring systemic treatment in the past 2 years
No history of autoimmune diseases	Immunodeficiency, or receipt of systemic steroid therapy or immunosuppressive therapy within 7 days of the first dose of the study drug
Adequent organ function as below:	Tumor infiltration to any important blood vessels and nerves
Absolute neutrophil count ≥1500/mm^3^	Concurrent other kinds of chemotherapy, targeted therapy, hormone therapy, immunotherapy, radiotherapy (except local symptomatic radiotherapy) or traditional Chinese medicine during the trial course
Platelet count ≥80,000/mm3	History of chemotherapy or immune checkpoint inhibitors
Haemoglobin ≥9.0 g/dL	Patients with serious complications, such as:
Total bilirubin ≤2 × ULN	Uncontrollable cardiovascular disease, angina and arrhythmia
Aspartate aminotransferase ≤3 × ULN (≤ 5 × ULN in patients with liver metastases)	History of myocardial infarction
Alanine aminotransferase ≤3 × ULN (≤ 5 × ULN in patients with liver metastases)	History of hemorrage or thromboembolic events within the last 6 months
Child-Pugh score ≤ 7	Uncontrollable diabetes mellitus or hypertension
Uric acid< 500 μmol/L	Uncontrolled intestinal lung disease or pulmonary fibrosis
Serum creatinine ≤1.7 mg/dL	Other patients who are considered to be unsuitable for this study by the investigator
Creatinine clearance ≥60 mL/min	
Proteinuria ≤2 g/24 h	
QTc interval ≤ 480 ms in ECG	
Written informed consent	

*Abbreviations*: *ULN* Upper Limit Of Normal, *ECOG PS* Eastern Cooperative Oncology Group performance status, *ECG* Electrocardiograph

### The course of the trial

**Fig. 1:**
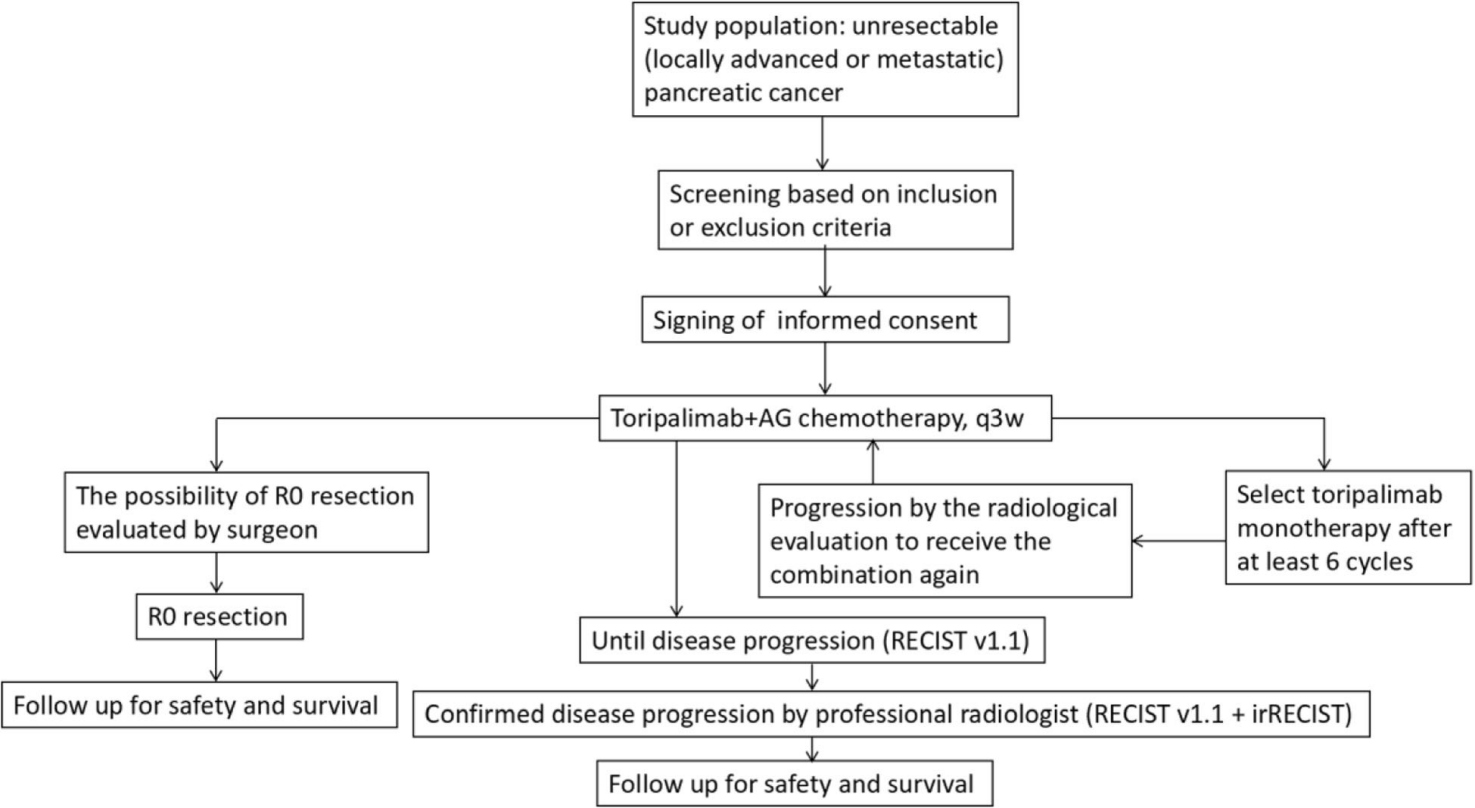
The main process of this clinical trial. Abbreviation: AG chemotherapy, nab-paclitaxel plus gemcitabine; RECIST, Response Evaluation Criteria in Solid Tumors. irRECIST, the immune-related RECIST

The main process of the trial is summarized in Fig. 1. Patients diagnosed histopathologically as PDAC and confirmed by the surgeon or MDT group as unresectable are included in this study. The entire course of the trial is expected to last more than 24 months. The subjects may discontinue the treatment because of progression disease (PD), intolerable toxicities, and requirements of patients or researchers. In addition, for the patients who complete 6 cycles of the combination therapy, the subsequent maintenance of toripalimab monotherapy is considered according to the patients’ response and tolerance to the treatment as well as the opinion of researchers. For the PD during the period of maintenance treatment, toripalimab combined with AG chemotherapy may be used again for systemic treatment. Pseudo progression possibly occurs during the immunotherapy, especially for patients during the maintenance therapy of toripalimab. Pseudo progression needs to be distinguished from true progression by the researchers, and the researchers need to determine whether to continue the therapy when pseudo progression is confirmed. After the end of treatment, the follow-up is conducted covering all patients to collect anti-tumor treatment information and OS.

After the first appearance of imaging evidence of PD evaluated by the Response Evaluation Criteria in Solid Tumors (RECIST) v1.1, its revised version, the immune-related RECIST (irRECIST), may be used to make treatment decisions according to tumor remission models of PD-1 blockades. For clinically stable patients with the first PD in imaging, the treatment may continue until the radiologist researcher reconfirm the PD after at least 4 weeks. When the PD is reconfirmed by the researchers or the radiologist researchers, the patients need to discontinue the treatment unless obtaining significant clinical benefits. Similarly, the evaluation of PD also needs to be reconfirmed by the whole research group.

For patients who are evaluated as partial response (PR), surgeons need to assess the surgical possibility. And for the part of patients who have the opportunity to receive R0 resection, the researchers need to communicate with the patients about the necessity of operation and guarantee operation only for patients without surgical contraindications. In fact, the feasibility of surgical resection needs to be considered during the whole therapy course by the surgeons. The patients who successfully underwent R0 resection also need close follow-up for safety and survival.

The possible AEs throughout the trial need to be monitored and graded according to the conventional term criteria for adverse events (CTCAE) version 4.0. Severe adverse events (SAEs) occurred within 90 days after the end of treatment need to be recorded. If the patients start new treatment, the AEs within 30 days need to be recorded.

**Fig. 2:**
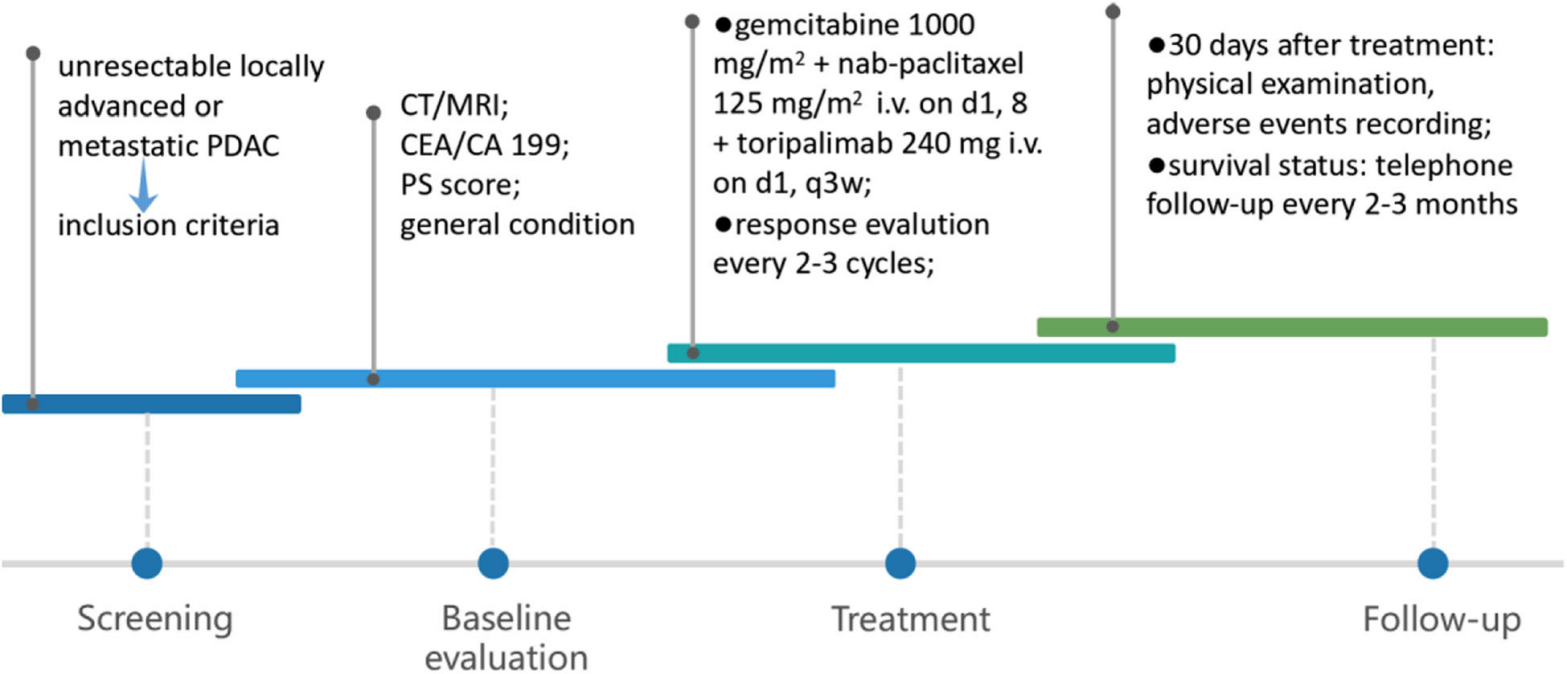
General overview of the course of the trial. The trial consists of four phases: Screening, aiming at verification of patients’ eligibility for the trial; Baseline evaluation, obtaining the basic information about the disease before treatment; Treatment, in which the combination therapy is administered and the response evaluation and toxicities are investigated; Follow-up, observing the long-term safety and clinical efficacy of this strategy. Abbreviations: PDAC, pancreatic ductal adenocarcinoma

The trial course consists of four phases of screening, baseline evaluation, treatment, and survival follow-up **(**Fig. 2**).**

### Screening

The screening of patients, aiming at assessment of their eligibility for the trial, needs to be completed within 1 week prior to the initial of treatment. The comprehensive information of potentially eligible patients are collected and recorded. Necessary procedures during the screening include: sign of written informed consent, collection of demography and medical history, physical examination, the evaluation of PS score and the vital signs, and the test of clinical chemistry, hematology and coagulation, the liver and kidney function, cardiac biochemical markers and ECG. In the end, the inclusion and exclusion criteria will be reviewed and the final judgment on the subject’s eligibility will be made.

### Baseline evaluation

The evaluation for the baseline status needs to be implemented within 2 weeks before the first treatment. In this phase, the information specifically about the tumor needs to be collected as the baseline level. Related procedures including the test of tumor markers (CA19–9, CEA), imaging evaluation (CT or MRI) and the expression of MMR protein and PD-L1, the TMB level, the number of TILs, the absolute counts of CD3, CD4, and CD8 T cells, the diversity of TCR, the cloning of TCR in the tumor tissue, and the level of EBV-EAD-IgG or EBV-VCA-IgA.

### Treatment

Because the safe dose of toripalimab has been verified in other solid tumors, and nab-paclitaxel and gemcitabine both are standard chemotherapy agents for advanced pancreatic cancer, we intend to use a fixed-dose instead of the dose-escalation exploration in this study. The administration of the combined therapy is carried out as follows: gemcitabine 1000 mg/m^2^ and nab-paclitaxel 125 mg/m^2^ infused intravenously (i.v.) on day 1 and day 8, along with toripalimab 240 mg i.v. on day 1. Routine prophylactic anti-vomiting, liver protection and best support treatment should be initiated on day 1 of each cycle of chemotherapy, but other anti-tumor cytotoxic drugs, targeted drugs or research drugs should not be accepted at the same time. The necessities for patients to undergo palliative radiotherapy or operations to control local symptoms during the study were evaluated by investigator. All combined treatments should be recorded on the CRF form.

Tumor response and safety are evaluated every two or three 21-day cycles of treatment, including the test of tumor biomarkers and cardiac biochemical markers, as well as the CT or MRI. Within 1 week prior to every repeated therapy cycle, physical examination, conventional laboratory analyses and tolerance assessment of the previous cycle of treatment according to NCI-CTCAE v 4.0 are regularly performed. The similar physical examination and efficacy or safety evaluation are also indispensable for the patients who are approved for discontinuing this trial. Besides, the information about AEs and treatment discontinuation need to be recorded and the cause of death is indicated in case of death.

### Follow-up

During the follow-up phase, which begins from 30 days after the end of treatment to patients’ death, acute or late toxicities and therapeutic efficacy of this combination therapy will be assessed by the indicators described above. If no complication occurs, detailed survival status and subsequent anti-tumor treatment of patients are collected through telephone follow-up every 2 or 3 months.

### Outcome measures

#### Clinical efficacy assessment

The following indicators are needed for the clinical efficacy assessment:

1. The objective response (OR): According to the RECIST 1.1, OR is divided into the complete response (CR), partial response (PR), stable disease (SD) and progression disease (PD). In this trial, the ORR is defined as CR + PR, and CR + PR + SD is calculated as DCR. The size of the lesions (imaging and physical evaluation) must be measured using the same way throughout the trial for the accuracy of comparative results. The 95% confidence intervals of the ORR and DCR are calculated.
2. The survival outcomes: PFS is defined as the time from initiation of treatment to the first confirmed PD or cancer-related death. OS is the time from the initiation of treatment to the death of patients. Kaplane-Meier analysis is used to estimate and determine for PFS and OS.
3. The resection rate is defined as the percentage of initially unresectable patients who successfully received the operation after at least one cycle of AG plus toripalimab;
4. The R0 resection rate is defined as the percentage of initially unresectable patients who successfully received the R0 resection after at least one cycle of AG plus toripalimab.

### Pharmacodynamic analysis

Fisher precise test is used to analyze the correlation between ORR and these potential pharmacodynamic parameters (such as MMR status, PD-L1 expression, TIL number, the cloning of TCR in TIL, T cell subsets in peripheral blood and the diversity of TCRs in peripheral blood). The frequency distribution diagram of tumor response and the curve of biomarkers levels are portrayed when there exists a correlation. The Cox proportional hazard regression analysis is used to investigate the relationship between tumor response and biomarkers, respectively.

### Safety assessment

The safety assessment includes observation and recording of any grade of AEs and SAEs during the therapy course, as well as the results of laboratory analyses and the ECG, physical examination and PS score, etc. AEs are graded according to NCI-CTCAE 4.0. The researchers are responsible to take appropriate measures for the AES and determine the causal relationships between the adverse events and the experimental drugs.

### Statistical considerations

#### Estimated number of enrollments

The sample size of this trial is estimated using curative effects as the estimation index. According to the Simon two-phase method, alpha equals 0.05 (both sides) and beta equals 0.2. On the basis of the 12-month OS rate (35%) in the MPACT study, the 12-month OS rate of this trial is expected to reach 55%. Therefore, 17 patients are enrolled in phase I, and if one-year OS is less than 6/17, which means less than six patients survived more than a year, the trial will be suspended. To the contrary, if the one-year OS is more than 6/17, 32 patients will be enrolled in phase II. In total, this trial intends to enroll at least 49 patients and the target sample size is 54 considering the 10% of patients may be lost to follow-up.

## Discussion

This trial is the first research to investigate the benefit and toxicity of the triple combination of toripalimab and AG for treatment-naive patients with unresectable PDAC. Toripalimab is a novel PD-1 antibody developed in China and is approved for refractory metastatic melanoma. It has a high binding affinity, which enables it to bind its specific antigen PD-1 receptor more firmly and compete better with PD-L1 and PD-L2 binding on tumor cells. Given its excellent safety, toripalimab may act as a backbone and combine with other systemic treatments to improve the response to ICIs [19]. Preclinical evidence supports the synergistic function between ICIs and chemotherapy. For instance, chemotherapy is considered to prime the “cold” immuno-environment of PDAC through increasing the expression of neoantigens or limiting immunosuppression, thus synergistically enhancing the anti-tumor immune response of ICIs [20]. Some phase I/II studies have confirmed the improvement of anti-tumor efficacy of chemotherapy combined with ICIs in various solid tumors [5, 21, 22]. The combination of toripalimab plus capecitabine and oxaliplatin was proved to effective and tolerable for patients with advanced gastric cancer, with the ORR of 66.7%, the DCR of 88.9%, and the SAEs incidence of 38.9% [23]. However, limited data about the combination of ICIs and chemotherapy against PDAC, with the ORR ranging from 14 to 80%. Therefore, the concurrent treatment of toripalimab plus AG chemotherapy represents a potential approach and is necessarily investigated for unresectable PDAC patients.

Safety profiles are also an indispensable part investigated in this trial to affect the therapy selection of patients. For the unresectable patients who received the combination treatment of nivolumab plus AG chemotherapy, a study has demonstrated that the most common grade 3 or 4 AEs were anemia (33%) [24]. As a phase II trial indicated, the treatment-naive metastatic PDAC patients received the combination of four drugs, including AG chemotherapy plus doublet ICIs (durvalumab and tremelimumab), and the most frequent grade 3 or higher AEs were hypoalbuminemia (45%), abnormal lipase (45%) and anemia (36%) [25]. The rate of grade 3 or higher AEs of immuno-chemotherapy ranges from 33 to 76% according to previous research. Besides, for the initially unresectable patients who receiving the curative surgery, the neoadjuvant combination therapy induces impressive tumor response to transform “unresectable” to “resectable” and good safety to tolerate subsequent operation. Previous studies demonstrated that neoadjuvant nivolumab induced a major pathological response in order to completely resected [26]. Neoadjuvant combination of pembrolizumab, capecitabine, and radiotherapy also had a manageable safety profile in PDAC without delaying the surgery [27].

In addition, the biomarkers that are suggested to predict the sensitivity of ICIs in other types of tumors, such as MMR deficiency, MSI status and TMB, are identified relatively rare in PDAC patients. For example, MMR deficiency is identified occurring at a frequency of 0.8% [28], meanwhile, MSI status is found in 2% of pancreatic patients. The investigation about novel potential biomarkers for predicting the response to PD-1 blockades in PDAC is desperately needed. Increasing analyses showed the potential value of PD-L1 overexpression to select a wider population to benefit from the PD-1 blockades [29, 30]. In advanced gastric cancer, pembrolizumab showed a higher ORR in patients with positive PD-L1 expression than non-selected patients [31]. In the patients with PD-1 positive expression (≥50%), pembrolizumab was proved to induce a significantly better PFS, therefore being approved as a first-line treatment for metastatic NSCLC patients with PD-1 overexpression (≥50%) [11]. Nevertheless, its application in PDAC has yet to be validated. TILs are the effector immune cell to directly affect the immune response. The subsets of TIL, the ratio of CD3+, CD8+, and granular enzyme B(GZMB) + T cells are regarded as predictive parameters in colon cancer [32]. The significant connection of TILs and the response to ICIs is proved in melanoma [33]. However, in PDAC patients, the expression of TIL and PD-L1 may be insufficient to predict the clinical outcomes of neoadjuvant GVAX vaccination [34]. Moreover, the underlying viral infection is suggested to be more sensitive to PD-1 blockades. A recent study found a 100% response to pembrolizumab in six EBV-positive patients with advanced gastric cancer [35]. Overall, a comprehensive landscape of tumor immunoenviroment, including a set of indicators, may be fully qualified for selecting the optimal treatment for individual patients.

In conclusion, this trial is the first attempt to evaluate the efficacy and safety of the combination of toripalimab plus AG chemotherapy as a novel first-line selection for unresectable PDAC patients. The results of this phase Ib/II study will provide preliminary supports for further assessment of this combined therapeutic regimen for unresectable PDAC patients.

## Data Availability

The datasets used and analyzed during the current study are available from the corresponding authors on reasonable request.

## Abbreviations

AEs: Adverse events
AG: Nab-paclitaxel plus gemcitabine
CTCAE: Conventional term criteria for adverse events
DCR: Disease control rate
EAD: Early antigen
EBV: Epstein-Barr Virus
ECG: Electrocardiograph
ECOG PS: Eastern Cooperative Oncology Group performance status
ICIs: Immune checkpoint inhibitors
irRECIST: The immune-related RECIST
MMR: Mismatch repair
MSI: Microsatellite instability
NSCLC: Non-small cell lung cancer
ORR: Objective response rate
OS: Overall survival
PD: Progression disease
PD-1: Programmed death-1
PDAC: Pancreatic ductal adenocarcinoma
PD-L1: Programmed death ligand-1
PFS: Progression-free survival
PR: Partial response
RECIST: Response Evaluation Criteria in Solid Tumors
SAEs: Severe adverse events
TCR: T cell receptors
TMB: Tumor mutation burden
TILs: Tumor-infiltrating lymphocytes
ULN: Upper limit of normal
VCA: Viral capsid antigen
